# Dynamic touch for embodying teacher's verbal instruction: Implications from classical ballet

**DOI:** 10.3389/fpsyg.2022.1067658

**Published:** 2022-12-22

**Authors:** Akito Miura, Haruka Seki

**Affiliations:** ^1^Faculty of Human Sciences, Waseda University, Saitama, Japan; ^2^Japan Ballet Educational Association, Tokyo, Japan

**Keywords:** ballet education, dance, effortful touch, embodied cognition, teaching method

## 1. Introduction

Teaching a motor skill in physical education involves the teacher's visual and verbal demonstration (Ryan et al., 2016). This often accompanies physical contact from the teacher to the learner (Choi and Kim, 2015). For example, imagine a classical ballet lesson where the teacher teaches the beginner learner how to stand correctly. First, the teacher stands correctly and verbally explains how to position the body. Next, the teacher touches the learner's body and corrects their alignment and form. When teaching other techniques, some teachers push the body part of the moving learner and change their movement trajectory. Thus, for teaching motor skills, touch often occurs from the teacher to the learner, and its primary role can be described as a mechanical effect on the learner's body.

In this article, however, we contend that touch has cognitive significance for learners, that is, the embodiment of the teacher's words from the perspective of cognitive and movement sciences. Among many physical contacts, we will focus specifically on learner-to-teacher touch, for example, when the learner pushes the teacher's arm to ascertain the stiffness of the joints. We discuss that without it, the problem of the symbol merry-go-round is likely to occur.

## 2. Symbol merry-go-round in ballet lessons

Learners (especially beginner learners) often encounter several problems in ballet lessons. Learners must learn how to subtly control muscle tension and engagement levels; however, for several reasons, it is difficult to estimate the state of muscle activity by watching the teacher's kinematics (e.g., the trajectory of movement, velocity, and acceleration). First, the adjustments in the level of muscle activity do not always accompany kinematic changes. For example, when adjusting poses or techniques for expressive nuances (e.g., lightness vs. strength), the kinematics do not change much, but muscle tension and engagement levels do. Second, the skill requirement of ballet is high. Warren and Cook (1989, p. 30) state, “The hand must appear relaxed at all times.” Note that they do not say that the hands must always be relaxed, instead, it must always “appear” to be so. This requirement implies that the better the teacher's movements are during the ballet, the more confused the learner will be about the muscle tension and engagement levels.

Moreover, as the level of muscle tension and engagement is difficult to convey through kinematics, the teacher is compelled to describe it verbally. This is when the problem of the symbol merry-go-round (Harnad, 1990) is likely to arise. This problem has been pointed out in embodied cognitive science, which regards human cognition as being formatted in sensorimotor experience (and the neural systems used for it) rather than independent of the body and behavior (Glenberg, 2015; Fincher-Kiefer, 2019). Harnad (1990) and Glenberg (2015) illustrate this problem with the example of learning Chinese from a Chinese/Chinese dictionary. Imagine that someone who does not speak Chinese lands at an airport in China and would like to know the meaning of a Chinese word on a signboard. They look up the meaning in their Chinese/Chinese dictionary; however, the word is rephrased in other Chinese words, and they have to search further for its meaning in the dictionary. However, repeating this makes no sense to them, as the symbols are simply rephrased. This means that symbols divorced from something outside the symbol system (e.g., sensorimotor experience) do not provide meaning.

Consider a specific example where you will understand the word “love” only using an English/English dictionary. In the dictionary (Merriam-Webster, n.d.), love is defined as “strong affection for another arising out of kinship or personal ties”. Then, you search “affection” and find that it is defined as “a feeling of liking and caring for someone or something”. No matter how often you repeat this process, it will not lead to an understanding of the meaning of love, unless some words in the dictionary are connected to your sensorimotor experience.

Fincher-Kiefer (2019, p. 84) points out the possibility that experts have a rich sensorimotor experience of their skills. Therefore, the language they use to express these skills are more embodied than in a novice. This suggests that the more experienced the ballet teachers are, the richer variations of embodied words they use in the lesson. Beginner ballet learners may receive many words from their teachers, but the words can easily cause a symbol merry-go-round because learners may have little bodily experience associated with them. Conversely, beginner learners may require relevant sensorimotor experiences to embody the teacher's verbal instruction.

## 3. Embodiment by touch

### 3.1. Language and bodily experience

We propose learner-to-teacher touching in a way that is called “dynamic (effortful) touch” in ecological psychology (Gibson, 1966; Turvey and Carello, 2011; Carello and Turvey, 2016) as a means of stepping off the symbol merry-go-round. Dynamic touch involves moving an object dynamically to determine its physical properties. For example, to ascertain the weight of something, we can toss it, receive it, or shake it in our hands to perceive the weight more accurately than by simply holding it (Gibson, 1966). Our proposal for learners is to dynamically and gently move the teacher's body to embody their instruction. The ballet teacher could, for example, demonstrate a defined arm posture and allow the learner to manipulate their forearm to explore muscle tension.

To provide a rationale for our proposal, we introduce the behavioral and neuroscientific evidence showing that language comprehension and bodily experience are related (for a systematic understanding, please see Fincher-Kiefer, 2019, chapter 5). For example, Glenberg and Kaschak (2002) reported that comprehending a sentence that describes an action in one direction (e.g., “close the drawer” means the direction away from the body) is facilitated by hand movement that is compatible with that action. This is known as the action–sentence compatibility effect (ACE)^1^. Studies on children have shown that physically manipulating objects related to the reading material enhances their reading comprehension (Glenberg et al., 2004, 2007). A neuroimaging study demonstrated that while one processes words referring to face, arm, and leg actions (to lick, kick, and pick), the brain regions that are active during actual actions of those body parts activate (Hauk et al., 2004).

Kontra et al. (2015) reported interesting evidence that bodily experience (i.e., the dynamic touch of an object) deepens the understanding of physics concepts. They tested whether the understanding of physics concepts (torque and angular momentum) is facilitated by bodily experience. In their experiment, participants either (1) held and moved a device made from bicycle wheels to experience torque and angular momentum or (2) observed someone doing it. They found that the participants who moved the device understood the physics concepts better (obtained a better score in the post-test) than those who observed it. In addition, the follow-up neuroimaging experiment revealed that the better score was explained by the activation of sensorimotor brain regions when they reasoned about angular momentum.

The aforementioned studies show that language is grounded in the body (i.e., embodied) and that actual touch facilitates the embodiment and deepens understanding of the subject. This suggests that it is essential for beginner ballet learners to touch the teacher dynamically to embody the teacher's words effectively.

### 3.2. Embodiment in ballet lesson

Let us explain what can be known by dynamic touch itself. As observed in many ballet skills, subtle control of muscle tension and engagement, while the kinematics do not change significantly, can be regarded (although not exclusively) as adjusting the level of muscle cocontraction. Cocontraction refers to a situation where the agonist and antagonist muscles are active simultaneously (Latash, 2018). The level of cocontraction corresponds to the level of joint stiffness, defined as a ratio of the force applied to a joint from outside to an angle at which it is flexed or extended. Increased cocontraction results in increased joint stiffness because the muscles firmly pull the bone from both sides of the joint.

It is difficult to determine the level of cocontraction from the kinematics because one can change the level of cocontraction without changing the kinematics. However, joint stiffness can be perceived by dynamically moving the joint from the outside, meaning that the level of cocontraction can also be perceived by dynamic touch.

As a practical example, the following approach may be helpful. Let us consider the case of embodying the teacher's verbal description of the muscle tension and engagement level of arms such as during poses. First, the teacher reproduces the level of muscle tension of the arm as good and bad examples. Then, the learners hold the forearm of the teacher with one hand firmly and try to push, pull, or sway it in various directions. Any touch should be forceful enough to move the joints of the person being touched. When swaying the arms, the learners should sway them rhythmically to the extent that they move at least a few centimeters. By doing this, the learners can learn about the appropriate cocontraction level around the teacher's elbow and shoulder joints. Comparing good and bad examples will help one to better understand the correct level. The teacher's verbal description corresponds with the learner's sensorimotor experience through dynamic touch, allowing the teacher's words to become embodied in the learner. The same approach can be applied to the legs during poses such as an arabesque or retire.

We do not recommend touching a moving body such as in turns because it can be dangerous. Experienced ballet teachers can reproduce the stiffness of the arms and legs in turns (e.g., pirouette and piqué turns) while standing still without actual rotation. We recommend that the learner touch them dynamically.

## 4. Discussion

Although this article covered a limited situation in ballet lessons, it has provided an essential perspective for beginner learners to step off the symbol merry-go-round and embody the teacher's words by dynamic touch. We consider this to be significant in two ways. First, while the positive effects of physical contact in dance pedagogy have been often discussed (Assandri, 2019; Hermans, 2021), the use of dynamic touch from learner to teacher has not been discussed. This may be partly because touching teachers is culturally and socially discouraged. However, learner-to-teacher touch has some substantial benefits for skill learning, as explained in this article. How dance educators can incorporate this into the field must be thoroughly discussed elsewhere.

Note that the learner-to-teacher dynamic touch is not the only solution for the symbol merry-go-round problem. The teacher-to-learner touch can also act on the learner's sensorimotor experience. However, dynamic touch is significant in enabling active and exploratory information acquisition. In ballet lessons, because teachers tend to give information unilaterally to the learner, active information acquisition is considered essential from a pedagogical perspective (Culp et al., 2020). Active learning in physical education needs further study, and this article provides insight into it.

## Author contributions

All authors listed have made a substantial, direct, and intellectual contribution to the work and approved it for publication.

## Footnotes

^1^There is controversy over the reliability of some versions of the Glenberg and Kaschak ACE, most notably Morey et al. (2022). Nonetheless, a recent meta-analysis (Winter et al., 2022) concludes that the effect is small but reliable.
